# Sensitivity of photoelectron diffraction to conformational changes of adsorbed molecules: Tetra-tert-butyl-azobenzene/Au(111)

**DOI:** 10.1063/1.4975594

**Published:** 2017-02-01

**Authors:** A. Schuler, M. Greif, A. P. Seitsonen, G. Mette, L. Castiglioni, J. Osterwalder, M. Hengsberger

**Affiliations:** 1 Physik-Institut, Universität Zürich, Winterthurerstrasse 190, 8057 Zürich, Switzerland; 2 Département de Chimie, Ecole Normale Surpérieure, 24, Rue Lhomond, 75005 Paris, France; 3 Fachbereich Physik, Philipps-Universität Marburg, Renthof 5, 35032 Marburg, Germany

## Abstract

Electron diffraction is a standard tool to investigate the atomic structure of surfaces, interfaces, and adsorbate systems. In particular, photoelectron diffraction is a promising candidate for real-time studies of structural dynamics combining the ultimate time resolution of optical pulses and the high scattering cross-sections for electrons. In view of future time-resolved experiments from molecular layers, we studied the sensitivity of photoelectron diffraction to conformational changes of only a small fraction of molecules in a monolayer adsorbed on a metallic substrate. 3,3′,5,5′-tetra-tert-butyl-azobenzene served as test case. This molecule can be switched between two isomers, *trans* and *cis*, by absorption of ultraviolet light. X-ray photoelectron diffraction patterns were recorded from tetra-tert-butyl-azobenzene/Au(111) in thermal equilibrium at room temperature and compared to patterns taken in the photostationary state obtained by exposing the surface to radiation from a high-intensity helium discharge lamp. Difference patterns were simulated by means of multiple-scattering calculations, which allowed us to determine the fraction of molecules that underwent isomerization.

## I. INTRODUCTION

Small organic molecules that undergo pronounced and reversible conformational changes upon photoexcitation represent an important class of molecular switches. They can be used as building blocks in materials and devices with promise for interesting optoelectronic and optomechanical applications.^1–3^ Adsorbed on surfaces, they are of interest in the context of molecular electronics and smart surfaces where they may introduce functionalities that can be controlled by light of specific wavelengths. For this purpose, one would like to characterize their switching behavior in the adsorbed state in terms of structural rearrangements and structural dynamics.

The photo-induced *trans* to *cis* isomerization in azobenzene along the N = N double bond connecting the two phenyl rings is an early example of photo-switching^4,5^ that has been studied in great detail in solution later on.^6^ Azobenzene was found to adsorb on Au(111) in well ordered monolayers.^7^ However, the photo-switching ability of the molecule is not preserved, most likely due to the presence of short-lived electronic states in the metal substrate, which dissipate energy before the conformational change is finished.^8^ The coupling to the surface can be reduced by the attachment of four tert-butyl (TB) ligands to the 3 and 5 positions of the two phenyl groups of the azobenzene molecule. These spacers allow the molecule to maintain the switching ability on a metal surface. Indeed, the light-induced *trans-cis* isomerization of tetra-tert-butyl-azobenzene (TBA) adsorbed on Au(111) has been observed by scanning tunneling microscopy (STM)^8^ and by two-photon photoemission spectroscopy (2PPE).^9^

**FIG. 1.**
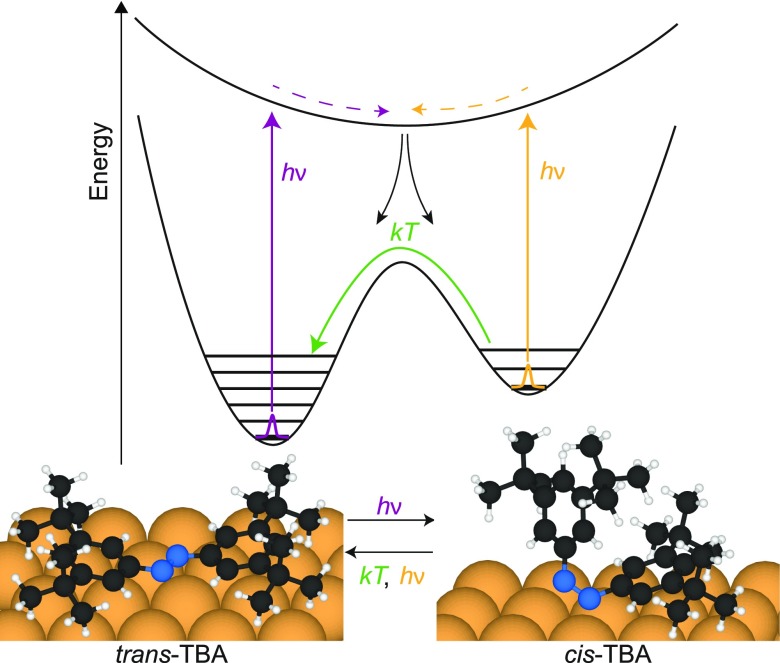
Schematic diagram of the photoisomerization process: TBA adsorbed on a Au(111) surface exhibits two different structural configurations, *trans* and *cis*. Since the energy minimum of the *cis* isomer is significantly more shallow than the one of the *trans* isomer, the net thermal transition rate drives the molecule towards the *trans* state.^17^ The molecule can be switched between *trans* and *cis* by absorption of light as depicted by the dashed arrows.^9^

The pronounced structural changes in the TBA/Au(111) system associated with the *trans-cis* isomerization have been studied experimentally by means of near-edge x-ray absorption fine structure (NEXAFS)^10^ and theoretically by density functional theory (DFT).^11^ The *trans* isomer adsorbs in a nearly planar geometry with both phenyl rings parallel to the surface. In the *cis* isomer, one of the phenyl groups flaps up by ≈30° while the second one finds itself perpendicular with respect to the surface,^10^ as shown in Fig. 1 (bottom part). In the present work, it is explored to what degree this structural rearrangement can be detected via x-ray photoelectron diffraction (XPD),^12^ which can in principle provide very detailed and precise information on adsorbate geometries.^13,14^ This is done in view of future time-resolved XPD experiments with femtosecond resolution in a pump-probe scheme^15,16^ that may shed light on the switching dynamics.

In XPD, an electron is promoted from a core-level to a continuum of free-electron-like states by absorption of an x-ray photon. The photoelectron wave propagates from the emitting atom through the surface and is partially scattered by neighboring atoms, thus generating an interference pattern by coherent superposition of direct and scattered waves.^12^ The angular intensity distribution is recorded as a function of emission direction. At kinetic energies of a few hundred eV, XPD patterns are dominated by relatively narrow forward focusing peaks.^18^ These diffraction maxima are caused by small-angle scattering where the scattering form factor is strongly peaked. Thus, these maxima in the photoemission intensity distribution directly indicate real-space vectors between the emitter site and other atomic sites.

One particular challenge for time-resolved XPD studies of molecular switches will be that, for low pump fluence, only a small fraction of the adsorbed molecules may undergo the *trans-cis* isomerization and that the isomerization dynamics will follow many different trajectories. Since photoemission samples a macroscopic surface area, the XPD pattern represents a superposition of many coexisting conformations.^15^ The goal of the present work is to compare the XPD pattern from a monolayer of TBA molecules on Au(111) in thermal equilibrium at room temperature with a XPD pattern obtained from the same ensemble of molecules during exposition to ultraviolet (UV) radiation from a continuous high-intensity source. The differences in the patterns are related to conformational changes of the excited molecules. In combination with scattering calculations, the quantitative analysis of the XPD patterns allows the fraction of excited molecules to be determined.

The *trans* isomer is the stable ground state of the azobenzene molecule, with the *cis* isomer slightly higher in energy and separated by a high barrier in the potential energy surface (Fig. 1, top part). In solution, the absorption of ultraviolet (UV) light (≈365 nm) can promote it to the *cis* state via *π* → π* (*S*_2_) excitation. The reverse reaction can be triggered with high quantum yield by blue light (≈420 nm)^5,19^ by *n* → π* (*S*_1_) excitation. Depending on the excitation wavelength and light intensity, a so-called photostationary state (PSS) is established in an ensemble of azobenzene molecules with a defined *cis*-to-*trans* ratio. Adsorbed on a surface, the situation is different in that the absorption spectra change and any photon with energy >2 eV can trigger isomerization in either direction.^17^ It has been shown that the excitation proceeds predominantly via electron-hole pair creation in the metal substrate followed by a positive ion resonance due to charge transfer of holes from the substrate d-bands into the HOMO.^20^ A more detailed discussion can be found in Section IV.

## II. EXPERIMENT

### A. Data acquisition

The Au(111) surface was prepared by cycles of sputtering and annealing in order to obtain a clean and highly ordered surface. The surface quality was checked by means of x-ray and ultraviolet photoelectron spectroscopy (XPS and UPS, respectively) and low-energy electron diffraction. TBA was synthesized and characterized according to the procedure given by Alemani *et al.*^21^ The preparation and adsorption behaviour of TBA on Au(111) is discussed in detail by Hagen^22^ The molecules were evaporated onto the surface from TBA powder with a home-built Knudsen cell at a temperature of 370 K. The substrate was held at 410 K during deposition.^22^ The coverage of the molecular layer is important: In the compressed phase (1 ML = 1 monolayer), the molecular interactions are presumed to be stronger and the phenyl rings are tilted with respect to the surface whereas in the relaxed phase (coverages up to 0.9 ML) they lie parallel to the substrate for the *trans* isomer.^9^ The coverage was controlled during deposition by monitoring the workfunction change^23^ and after deposition determined by means of XPS. All data presented here were taken for 0.9 ML TBA/Au(111).

The photoemission data were recorded at room temperature and at a pressure of *p* < 3 × 10^−10^ mbar during the measurement without He lamp in a modified version of the VG Escalab 220 spectrometer.^24^ For the XPS/XPD measurements, photons from the Mg K*α* line (*hν* = 1253.6 eV) were used. The five-axis sample goniometer of the manipulator allows one to rotate the sample orientation in order to map the photoemission intensity distribution over the full hemisphere above the sample surface. Every angular setting, and thereby every data pixel represents emission into a solid angle of the same size. The data were background corrected by subtracting a background function slowly varying with the polar emission angle, and averaged taking advantage of the three-fold rotational symmetry of the substrate around the surface normal. For visualization, they are represented in stereographic projection. A more detailed description of the data treatment will be given in Section III.

A microwave-driven high-intensity He discharge source (VUV5000, Gammadata AB)^25^ with the monochromator set to zero order was used to expose the molecules to visible and near-UV radiation, which is dominated by lines at wavelengths of 388 nm, 402 nm, 447 nm, and 505 nm, respectively.^26^ Moreover, very strong emission is found at 58.4 nm (21.2 eV, He I), and two weaker lines lie at 53.7 nm and 30.4 nm in the vacuum ultraviolet (VUV).^25^ It is known from scanning tunneling microscopy (STM)^8,27^ and two-photon-photoemission (2PPE)^9,20^ measurements that prolonged exposure of molecules to UV radiation drives the TBA/Au system into a photostationary state. The ratio of *cis* and *trans* isomers in dynamical equilibrium, i.e., the PSS is given by the ratio of the cross-sections for the two photo-induced transitions and the thermal transition rate.^28^ The time needed to reach the PSS depends essentially on the total isomerization cross-section and on the photon flux. By comparing the photon flux and photon-energy dependent cross-sections from previous work^8,9,20^ with those from our He discharge lamp, we estimate the time required to drive the system into the dynamical equilibrium to be less than one hour (see the quantitative analysis presented below in Section IV). This was confirmed *a posteriori* by the observation that we switched on the He lamp about one hour before starting the actual measurement and obtained consistent results for the different runs and for the different angular settings used during the 10–12 h of data acquisition.

### B. Electron diffraction in atomic clusters (EDAC) calculations

**FIG. 2.**
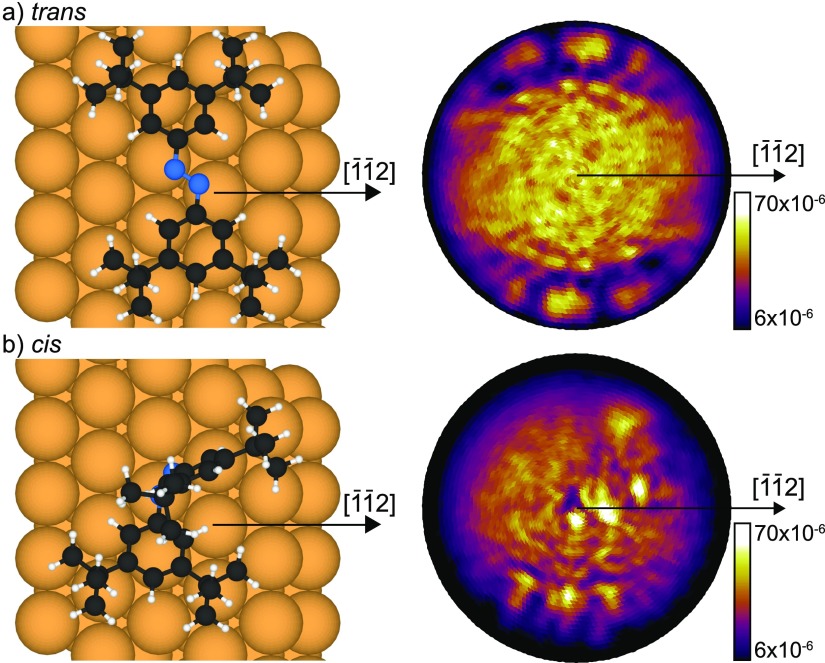
Hard sphere models and corresponding simulated scattering patterns of single TBA molecules in two different configurations: (a) *trans* isomer and (b) *cis* isomer. We assumed emission from the N 1s level using Mg-K*α* radiation (*E_kin_* ≈ 854 eV). The orientation of the substrate is the same, the horizontal corresponding to the direction $\left[\bar{1}\bar{1}2\right]$ as indicated. The adsorption geometries and the atomic positions were taken from McNellis *et al.*^11^

XPD patterns were simulated by multiple-scattering cluster calculations using the *electron diffraction in atomic clusters* (EDAC) code,^29^ which has been shown to describe experimental data well.^30^ Briefly, the calculations are based on interference of the direct wave and waves scattered several times by the atoms surrounding the emitter. The coherent sum is then evaluated at the position of detection in the far field. The computations are done by using a cluster model of the substrate, which allows adsorbed molecules to be included in a straightforward fashion.^14^ The atomic positions of the *trans* and *cis* isomers of TBA adsorbed on Au(111) were taken from the DFT calculation of McNellis *et al.*^11^ The two clusters consist of a single TBA molecule (*trans* and *cis*) on a gold (111) substrate. The total number of atoms in one cluster is *N* = 150. The atomic coordinates served as input to EDAC calculations, which are shown in Figs. 2(a) and 2(b) beside the atomic structures predicted by DFT. The kinetic energy for the simulations was 854 eV, which corresponds to emission from the N 1s core-level (binding energy of ≈400 eV) with photons from the Mg K*α* line (*hν* = 1253.6 eV).

## III. RESULTS AND QUALITATIVE ANALYSIS

The two simulated N 1s XPD patterns of Fig. 2 illustrate the pronounced differences that one would measure for an individual molecule that undergoes the *trans-cis* isomerization. At the kinetic energy of 854 eV, the XPD patterns are dominated by forward scattering (see the work of Greif *et al.*^14^ and references therein). As can be seen in Fig. 2(a), the dominant forward focusing peaks in the diffraction pattern for the nearly planar *trans* isomer appear near grazing emission and along azimuthal directions near ±90° away from the [$\bar{1}\bar{1}2$] direction. They correspond to forward focusing by the nearest carbon atoms, one for each nitrogen emitter. Carbon scatterers further away contribute less pronounced signals, because several of them lie within a narrow cone such that their scattering signals become strongly entangled. Intensities at lower emission angles arise due to higher order intramolecular scattering and backscattering from the substrate.

The *cis* isomer is far from planar since one of the phenyl moieties of the TBA is flapped up perpendicular to the surface while the second one is slightly tilted upwards^11^ (see Figs. 1 and 2(b)). Therefore, forward focusing signals now appear at angles closer to normal emission than for the *trans* isomer, forming a complex pattern with little symmetry. The brightest spots near the centre belong to the nearest carbon atoms of the phenyl ring, which is flapped up. The three less intense forward focusing maxima that can be found at higher emission angles near the bottom of the pattern are linked to the second, slightly tilted phenyl.

In order to demonstrate the sensitivity of the method to conformational changes of a small fraction of TBA molecules, we recorded two experimental XPD patterns for two different states: The first pattern was recorded without additional UV source (*trans* only). The second pattern was taken while the sample was illuminated with the He high intensity lamp with the monochromator in zero order. This led to a photostationary state with a specific ratio of *cis* to *trans* molecules.

**FIG. 3.**
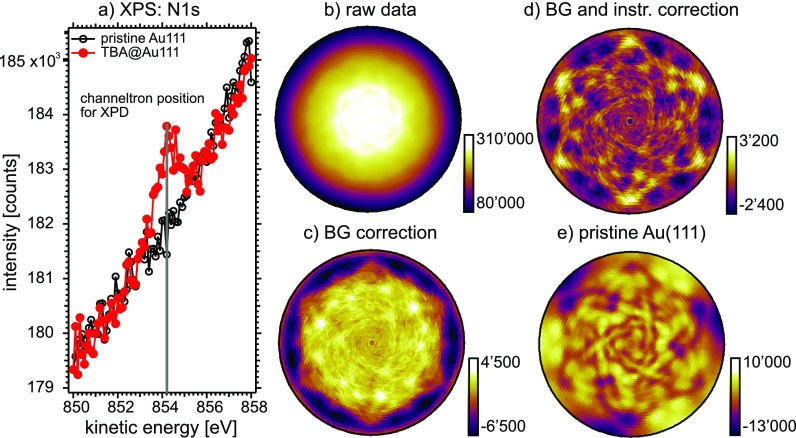
Data acquisition and data treatment: (a) Experimental XPS spectrum. The red peak is due to emission from the N 1s state. The intensity of this peak is recorded as a function of the emission angle which results in the raw XPD pattern (b). (c) N 1s XPD pattern after subtraction of the spectral background. (d) Final diffraction pattern after subtracting a Gaussian background in order to compensate for instrumental effects. (e) Spectral background measured for the same kinetic energy *E_kin_* ≈ 854 eV on the bare Au(111) surface.

The experimental procedure is visualized in Fig. 3 for a data set measured without UV illumination of the sample: The intensity of photoelectrons at the N 1s core-level position was recorded as a function of emission angle and displayed in stereographic projection, just like the EDAC simulations of Fig. 2. It is important to note that each pixel represents emission into a well-defined and constant solid angle. The raw data pattern (Fig. 3(b)) is essentially featureless except for a marked polar dependence. To make the intramolecular diffraction features visible, the spectral background underneath the N 1s peak was subtracted. This background was measured on a bare, clean Au(111) surface. It is not featureless but contains signatures of photoelectrons produced deep below the Au(111) surface as a consequence of multiple elastic and inelastic scattering within the fcc lattice.^31^ This particular way of background subtraction is not the standard procedure in XPD experiments, where normally the background is determined from intensity measurements to the left and right of the peak of interest and subsequent interpolation.^12^ In the present case, the standard procedure did not succeed mostly due to the very steep inelastic background (Fig. 3(a)). Finally, a two-dimensional Gaussian function centered at normal emission was subtracted from the data in order to remove polar dependencies related to instrument geometry. This includes the varying probe volume, x-ray spot size on the surface and changing inelastic attenuation length of the photoelectrons.^32^ Since we are interested in slight changes of the signal, only a minimum of data treatment shall be applied here. The same procedure was applied to the data taken with the sample being irradiated by UV light.

**FIG. 4.**
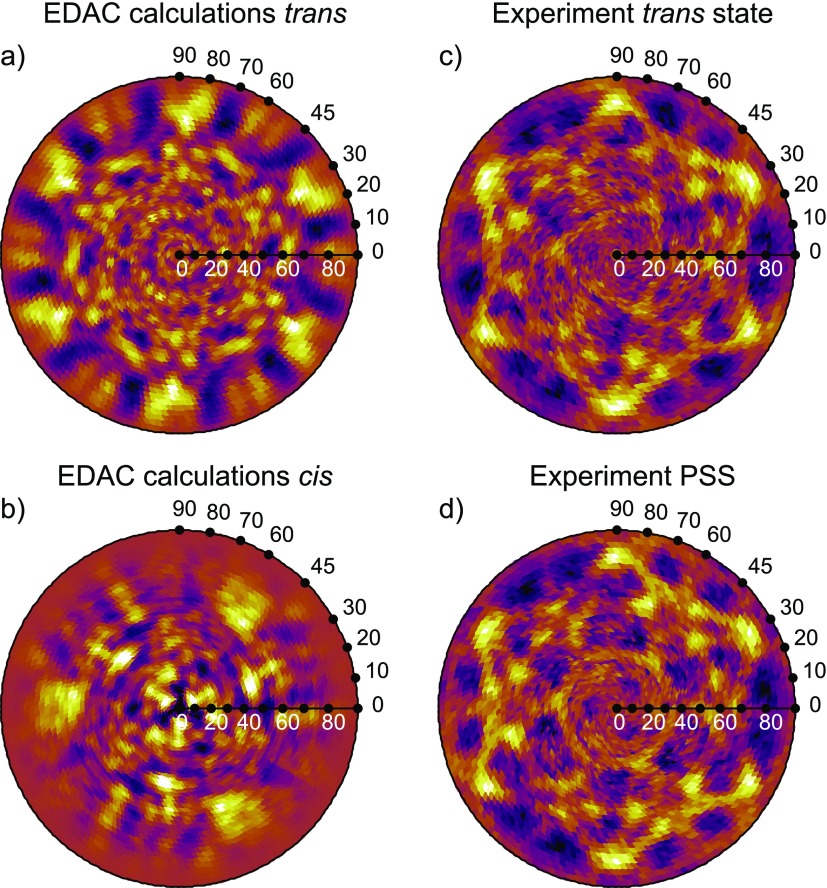
Comparison of simulated and experimental patterns: (a) and (b) EDAC calculations after three-fold averaging for *trans* and *cis* isomers, respectively. (c) and (d) Experimental XPD patterns for N 1s emission in *trans* and photostationary state, respectively.

When comparing the measured XPD data of Fig. 3(d), which should correspond to the *trans* isomer, to the EDAC simulations, one has to consider one more step: owing to the three-fold symmetry of the Au(111) surface, the simulated pattern needs to be symmetrized by summing up three patterns rotated by 120° with respect to each other. In Fig. 4, the experimental data are compared to the symmetrized results of the EDAC calculations. The agreement is good at large polar angles for the *trans* isomer: The six maxima related to nitrogen-carbon forward focusing between 70° and 80° appearing in the EDAC calculation for the *trans* isomer (Fig. 4(a)) can also be found in the experimental pattern (Fig. 4(c)). Some of the finer structures seen in the experiment are also reproduced. Remaining discrepancies might call for a refinement of the structural parameters, but this is beyond the scope of this work.

The XPD data from the UV illuminated sample, which was in a photostationary state with so far unknown *cis:trans* ratio, are shown in Fig. 4(d). The pattern is very similar to the one from the non-illuminated sample, with very subtle changes. This suggests that the *cis:trans* ratio is rather small. Accordingly, none of the features of the symmetrized *cis* state pattern (Fig. 4(b)) can be clearly recognized.

**FIG. 5.**
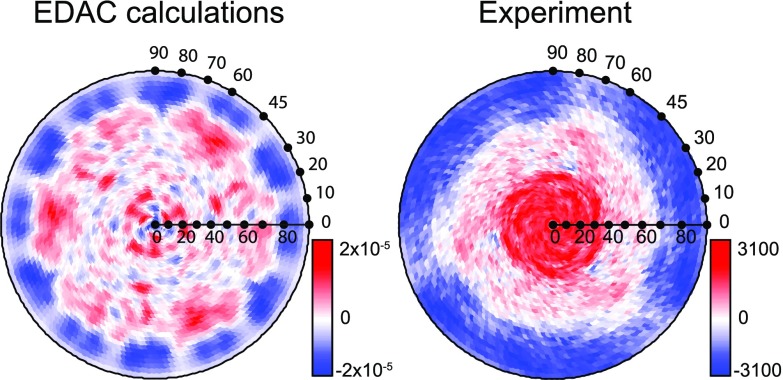
Difference patterns for the “*cis-trans*” isomerization. Left panel: EDAC simulation, right panel: difference of the two experimental patterns after three-fold average and normalization to the integral yield before background subtraction. The angular scales are indicated and the color code is defined such that blue color represents a loss of spectral weight and red color an increase of spectral weight upon excitation by UV light.

In order to verify that the signal changed upon illumination, we subtracted the raw data of the experimental patterns recorded in PSS and *trans* from each other after careful normalization for x-ray flux using reference measurements on the bare substrate. No background subtraction or correction for instrument functions was carried out because these effects affect both measurements in the same way and, therefore, cancel out in the difference pattern. Moreover, irrespective of the number of molecules that switched during the experiment, the difference pattern is always the same qualitatively if the total number of molecules is constant. The difference pattern is displayed in Fig. 5 and compared to the corresponding difference pattern obtained from the EDAC simulations.

Both the experimental and the simulated patterns show a clear shift of spectral weight from high to low polar angles. This can easily be understood by comparing the results with the structures of the isomers shown in Fig. 2. As described above, at kinetic energies of about 850 eV, the electrons are predominantly scattered in the forward direction. Hence, emission is enhanced at high polar angles for the *trans* isomer, and for lower polar angles in the *cis* isomer.

Moreover, some propeller-like lobes can be observed in the experimental difference pattern, which might correspond to three positive maxima found in the simulation at polar angles between 65° and 80° in Fig. 5. The structure in the experimental pattern appears to be broadened. Since in the *cis* isomer both phenyl moieties are lifted from the surface and since interactions between neighboring molecules are expected to be negligible, the phenyls may rotate around the nitrogen-carbon bonds, thus reducing strongly the rigidity of the molecules. As a consequence, any azimuthal structure, which leads to rather sharp features for emission from *trans* isomers, is expected to be smeared out in the case of the *cis* isomer, in agreement with our findings.

## IV. QUANTITATIVE ANALYSIS

A quantitative assessment is more complex. The main reason is that the calculation of the switching probability relies on the scattering calculations. In principle, one might compare the modulation amplitude in the difference pattern shown in Fig. 5 to the intensity of the pure *trans* signal, for instance. The EDAC calculations then provide the reference, i.e., the relative change in the pattern expected for the isomerization of 100% of the molecules on the surface. This procedure is prone to errors due to the instrumental normalization required to account for polar variations of the geometrical light incidence and analyzer focus and the inelastic attenuation length, as already outlined in Section III. In particular, any normalization procedure affects the distribution in polar angle. The latter is the most significant part of the difference patterns as can be easily seen in Fig. 5.

We have therefore chosen another approach to access the isomerization ratio quantitatively. We use [*cis*] and [*trans*] to denote the relative occurrences of *cis* and *trans* isomers on the surface with the normalization [*cis*] + [*trans*] = 1. We may then write the resulting photoelectron distribution in any state as follows:

$$I(\theta ,\phi )=[cis]\cdot I_{cis}(\theta ,\phi )+[trans]\cdot I_{trans}(\theta ,\phi ),$$ (1)

where *θ* and ϕ denote the polar angle with respect to the surface normal and the azimuthal angle around the surface normal with respect to the crystalline $\left[\bar{1}\;\bar{1}\;2\right]$ direction, respectively (see Figs. 2 and 4). We assume that the sample without UV illumination is close to pure *trans* state ([*trans*] = 1 and [*cis*] = 0), denoted hereafter as *I_trans_*, while the photostationary state can be written as mixed state *I*_PSS_. Since the concentrations [*cis*] and [*trans*] in *I*_PSS_ are still unknown, we test our assumption made in Eq. (1) and subtract the experimental *trans* pattern multiplied with a factor *x* ranging from 0 to 1 from the experimental pattern in the PSS

$$\Delta I(x,\theta ,\phi )=I_{\text{PSS}}(\theta ,\phi )-x\cdot I_{trans}(\theta ,\phi ).$$ (2)

**FIG. 6.**
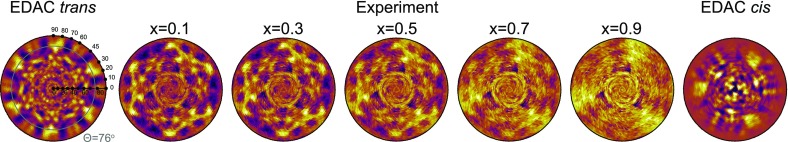
Comparison of calculated and experimental patterns for various values of *x*. From left to right: EDAC calculation for *trans*, experimental patterns $I_{\text{PSS}}(\theta ,\phi )-x\cdot I_{trans}(\theta ,\phi )$ for *x* = 0.1 through *x* = 0.9 and EDAC calculation for *cis*. The circle in the EDAC *trans* calculation indicates a polar emission angle of *θ* = 76°.

If we compare Eqs. (2) and (1), we see that at *x* = [*trans*] the resulting pattern ΔI(x,θ,ϕ) must be a pure *cis* pattern. This procedure is visualized in Fig. 6: starting at *x* = 0.1 with a pattern close to the calculated *trans* pattern and increasing *x* we continuously approach a pattern which is close to the EDAC simulation of the *cis* state. Note that as already mentioned above, the features are smeared out due to increased flexibility of the molecules around the nitrogen-carbon bonds.

**FIG. 7.**
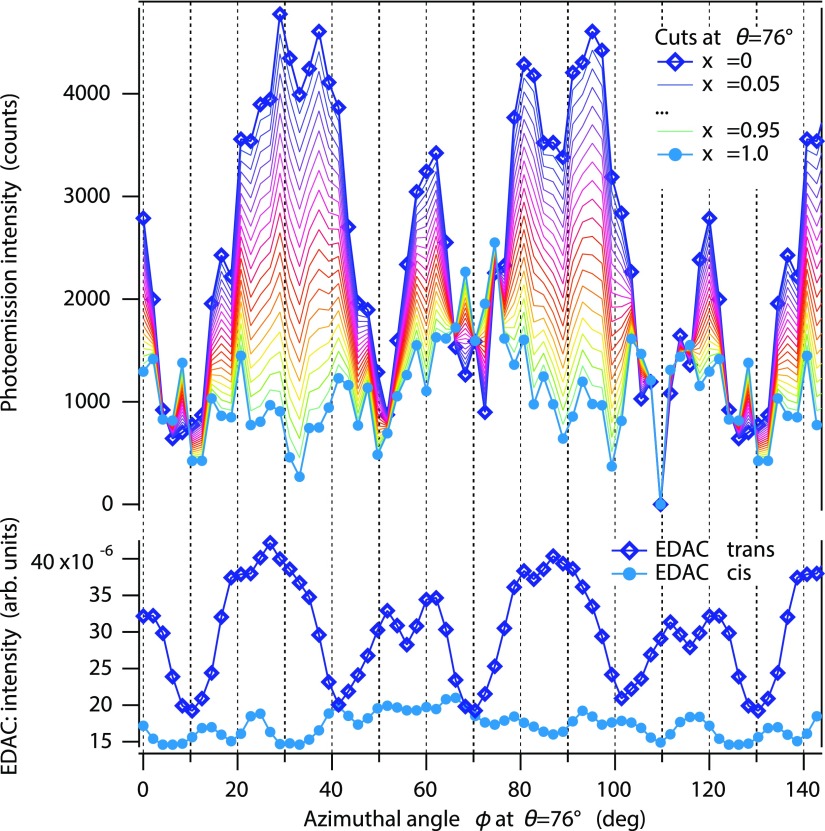
Azimuthal cuts at *θ* = 76° along the circle shown in Fig. 6. Top panel: experimental traces from *x* = 0 (topmost trace, blue open symbols) to *x* = 1.0 (bottommost trace, solid turquoise symbols) in steps of Δ*x* = 0.05 (see text for details). Bottom panel: azimuthal cuts through the simulated EDAC *trans* (blue open symbols) and *cis* (solid turquoise symbols) patterns at the same angular position.

The most clear signature of the transition from *trans* to *cis* is found at high polar angles. Therefore, we use these features as fingerprints and assume their intensity to be proportional to the density of the corresponding isomers. In addition, it is advantageous to compare traces at fixed polar angle: The intensity variation with polar angle is due to geometrical changes of the foci of light source and electron analyzer, and changes of electron escape depth, and optical matrix elements. The variation and the required normalization are *a priori* unknown. If only azimuthal rotations at constant polar angle are considered, these variations can be neglected. At *θ* ≈ 76°, we observe six sharp spots due to the well-defined nitrogen-carbon bonds in the *trans* state. They gradually disappear with increasing *x*. At *x* ≈ 0.9, three large lobes are observed and some weaker features reminiscent of the *cis* pattern. If we extract the intensity as a function of azimuthal angle at *θ* = 76° for all these distributions, we obtain the traces displayed in Fig. 7, which are compared to corresponding curves obtained from the calculations. One clearly observes the transition from the *trans* to the *cis* pattern by following the disappearance of the strong maxima at ϕ=30° and 90°.

For a more objective analysis, a statistical evaluation is required. In contrast to earlier studies (e.g., Refs. 33 and 34), in which a modified *χ*^2^ function was used,^35^ we used the Pearson correlation coefficient *r* to compare each of the azimuthal distributions shown in Fig. 7 with the corresponding curves from the *trans* and *cis* calculations. The Pearson *r*-factor is defined as the covariance of two functions normalized by the square roots of the variances of each of them.^36^ It does not require any treatment like scaling of the data prior to the calculation of the correlation function. In the case of maximum (anti-) correlation, *r* must approach the value of (–)1, a value of 0 corresponds to no correlation. We evaluated the following expression for *cis* and the analogous one for *trans*, and for the two most suitable angles *θ* = 76° and 78°,

$$\begin{array}{c} r_{cis}(x)=\frac{\sum_{i}\;\left(\Delta I(x,\theta ,\phi_{i})-\langle \Delta I\rangle \right)\;\cdot \;\left(I_{cis}(\theta ,\phi_{i})-\langle I_{cis}\rangle \right)}{s_{\Delta I}\;\cdot \;s_{I_{cis}}},\\ \text{with}\;s_{\Delta I}=\sqrt{\sum_{j}{\left(\Delta I(x,\theta ,\phi_{j})-\langle \Delta I\rangle \right)}^{2}},\\ \text{and}\;s_{I_{cis}}=\sqrt{\sum_{k}{\left(I_{cis}(\theta ,\phi_{k})-\langle \Delta I\rangle \right)}^{2}}, \end{array}$$ (3)

where ⟨ΔI⟩ and $\left\langle I_{cis}\right\rangle$ denote the average values of Δ*I* and *I_cis_* for all angles ϕ, respectively.

**FIG. 8.**
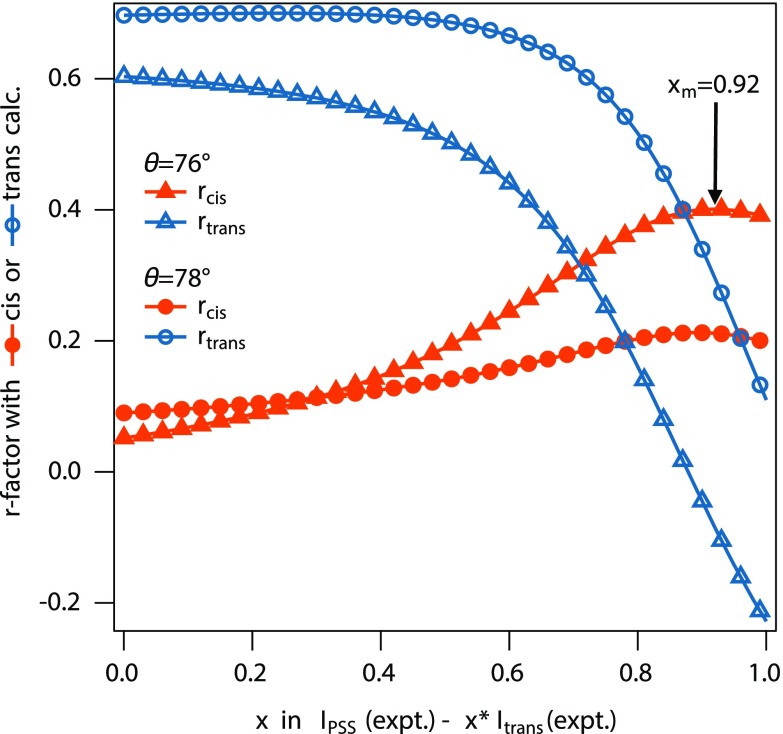
Pearson correlation coefficient^36^ *r* comparing the experimental cuts of Fig. 7 (*θ* = 76°, triangles) and for *θ* = 78° (circles) with the cuts through the calculated patterns for *cis* (solid orange symbols) and *trans* (open blue symbols) states.

The results are shown in Fig. 8. As expected for *x* ≈ 0, the *r* factor is higher for the *trans* state than for *cis*. When *x* increases the correlation with *trans* becomes weaker, while the *r*-factor reflecting the covariance with the *cis* trace increases. The *r*-factor of the *cis* goes through a maximum for both angles. The weighted mean value at which *r* is maximum is *x*_m_ = 0.92 ± 0.025. This means that 8.0% ± 2.5% of the molecules underwent isomerization into the *cis* state in our case.

In order to compare to published switching probabilities, we have to take the temperature and different photon energies into account. It must be emphasized that the isomerization process and the cross-sections change considerably when going from molecules in solution to molecules adsorbed on surfaces.^17^ Based on temperature- and photon-energy dependent 2PPE experiments, Hagen and co-workers proposed the following picture for the isomerization of TBA on Au(111):^17,20,28^ Electrons are excited by photons out of the substrate Au 5d manifold and the photohole is transferred to the HOMO of the TBA molecule. The minimum energy required for creating photoholes in the 5d levels is about 2.2 eV, which is in agreement with the observed threshold behaviour at 2 eV of the isomerization cross-section and the almost constant value for photon energies between 2.2 and 4.4 eV.^20^ For higher photon energies, the effective cross-section strongly increases again due to increased photoionization probability of Au 5d.

The transient positive ion resonance due to hole transfer into the HOMO allows for efficient energy transfer to the molecule and, thereby, triggers the isomerization. The isomerization may proceed in both ways, from *trans* to *cis* and from *cis* to *trans*. The branching ratio, i.e., the relative probabilities for the two directions are fairly constant in energy^17^ and roughly 1:1 slightly in favor of the *trans* to *cis* transition.^17,27^ While in solution the potential energy surface of TBA has two minima of comparable depth for the two isomers, the minimum of the *cis* isomer of TBA becomes shallow upon adsorption on a metal substrate. This favors thermally activated isomerization and leads to a net thermal isomerization rate from *cis* to *trans*. Thus, the relative fraction of *cis* and *trans* molecules is given by the ratio of the cross-sections for photo-induced switching and the thermal rate. The photostationary state comes closer to the pure *trans* state at higher temperature. The typical time scale for reaching the PSS is given by the effective isomerization cross-section and the photon flux.

While the VUV photon flux of the He discharge lamp used here is known from photocurrent measurements,^24,25^ the flux of visible and near-UV photons had to be determined in a separate experiment using a discharge source working at the same discharge pressure. We obtain a flux of about 1.1 × 10^14 ^ph/(cm^2^ s) for photon energies higher than 2.2 eV, about the same order of magnitude as the one of VUV photons of 1.8 × 10^14 ^ph/(cm^2^ s). Using these numbers and our experimental results, and assuming the known branching ratio^17^ for *cis* and *trans* isomerization, we achieve good agreement with observed values using an effective cross-section of the order of *σ*_eff_ ≈ 10^−17^ cm^2^. The time required to drive the system into the photostationary state, defined as 90% of the final *cis* concentration, is estimated to be about one hour, based on the results for the photon flux and cross-sections and in agreement with own experimental findings. The effective total isomerization cross-section *σ*_eff_ derived here is higher by several orders of magnitude than cross-sections of 10^−23^ cm^2^ at a photon energy of about 3 eV and 30 K (Ref. 27) or 10^−22^ cm^2^ for visible light and a temperature of 90 K.^28^ This can be explained by the higher photoionization cross-section of the Au 5d states^37^ and the possible direct photo-ionization of the TBA molecule.

## V. OUTLOOK: TIME-RESOLVED PHOTOELECTRON DIFFRACTION EXPERIMENTS

A standard photoelectron diffraction data set represents the angular distribution of photoemission intensity recorded at fixed electron kinetic energy for a chosen number of emission directions. When investigating sensitive surface systems like layers of organic molecules, data acquisition should be fast to avoid sample damage. Furthermore, the number of emission angles recorded will depend on the goal of experiment, because a full and accurate structure determination requires a fine grid in polar and azimuthal angles. According to experience, such a grid covering the full hemisphere above the sample has 4000–5000 angles, spaced such that each pixel represents the same solid angle. Using parallel data acquisition as it is possible with most modern electron detectors, the number of sample settings can be greatly reduced.^38^

The strategy will be different if the structural dynamics are studied. Time-resolved experiments are usually carried out in the pump-probe mode. After an excitation pulse (pump), the transient structure can be tracked by photoelectrons excited by a second time-delayed (x-ray) probe pulse. Thus, the time delay represents an additional parameter, which has to be varied, multiplying the number of measurements with the number of time delays. The latter is typically of the order of 100 steps. Our present results demonstrate the sensitivity of XPD to structural changes in a small number of molecules on top of a high background of molecules in the ground state. Such a measurement could correspond to one such time delay setting in a time-resolved pump-probe experiment. Realistically, however, the number of emission directions (pixels) is limited to the number of angular channels, which can be recorded in parallel in a single measurement. Longer integration times and, thereby, better data statistics often outweigh the number of pixels in such experiments.^35^

Quantitative assessment then requires the use of fingerprints representing all possible transient structures. Such fingerprints can be either numerical simulations as in our case here or the existence of reference data from these transient structures. Reference data must be taken from pure samples. This is impossible in the present case but such data can be measured in static measurements from all phases of a system undergoing a phase transition as a function of sample temperature, for instance. In order to compare the data, it turned out to be advantageous to analyze difference data. In this way, problems with data normalization due to a change in geometry upon sample rotation are minimized and subtraction of a background is not necessary because it cancels in the difference data to a good approximation. Moreover, in the present case the normalization was avoided by solely comparing traces as a function of the azimuthal angle at constant polar angle. Since the azimuthal rotation corresponds to a rotation around the surface normal, the experimental geometry does not change and any intensity modulations can be attributed to effects due to the electronic or atomic structure of the surface under investigation.^32^

The features of the patterns, which can clearly be attributed to one of the structures, can be used as fingerprints. In this case, a full structure determination at each delay is not required, which reduces considerably the measurement time. The intensity of the features is a simple measure of the number of molecules in the corresponding transient state. Statistical analysis can be used in order to assess the ratios of the isomers in a quantitative way. For each delay time step, the best match is found by evaluating reliability factors as shown in the present work.

## VI. CONCLUSION

In conclusion, we presented photoelectron diffraction data from tetra-tert-butyl-azobenzene molecules adsorbed on Au(111). Upon excitation with visible, ultraviolet, and vacuum-ultraviolet radiation, the molecules undergo reversible *trans*-to-*cis* isomerization, which can be detected in the diffraction patterns. Moreover, a quantitative analysis revealed that the isomerization can be driven very efficiently with high-energy vacuum-ultraviolet photons. This gives further evidence for an isomerization mechanism, which proceeds by generation of photoholes in the Au 5d shell and hole transfer into the HOMO, where a transient positive ion is formed. The excess energy is then used to drive the isomerization of the molecule.

The fact that a small number of switched molecules can be observed in photoelectron diffraction experiments on top of a large background of passive molecules opens new possibilities for studying structural dynamics in solid surfaces with potentially femtosecond time resolution.

## References

[1] M.-M. Russew and S. Hecht , “ Photoswitches: From molecules to materials,” Adv. Mater. , 3348 (2010). 10.1002/adma.200904102 20422653

[2] T. Hugel , N. B. Holland , A. Cattani , L. Moroder , M. Seitz , and H. E. Gaub , “ Single-molecule optomechanical cycle,” Science , 1103 (2002). 10.1126/science.1069856 12004125

[3] C. Zhang , M.-H. Du , H.-P. Cheng , X.-G. Zhang , A. E. Roitberg , and J. L. Krause , “ Coherent electron transport through an azobenzene molecule: a light-driven molecular switch,” Phys. Rev. Lett. , 158301 (2004). 10.1103/PhysRevLett.92.158301 15169322

[4] G. Hartley , “ The Cis-form of Azobenzene,” Nature , 281 (1937). 10.1038/140281a0

[5] G. Zimmerman , L.-Y. Chow , and U.-J. Paik , “ The photochemical isomerization of azobenzene,” J. Am. Chem. Soc. , 3528 (1958). 10.1021/ja01547a010

[6] P. Hamm , S. M. Ohline , and W. Zinth , “ Vibrational cooling after ultrafast photoisomerization of azobenzene measured by femtosecond infrared spectroscopy,” J. Chem. Phys. , 519 (1997). 10.1063/1.473392

[7] M. J. Comstock , J. Cho , A. Kirakosian , and M. F. Crommie , “ Manipulation of azobenzene molecules on Au(111) using scanning tunneling microscopy,” Phys. Rev. B , 153414 (2005). 10.1103/PhysRevB.72.153414

[8] M. J. Comstock , N. Levy , A. Kirakosian , J. Cho , F. Lauterwasser , J. H. Harvey , D. A. Strubbe , J. M. J. Fréchet , D. Trauner , S. G. Louie , and M. F. Crommie , “ Reversible photomechanical switching of individual engineered molecules at a metallic surface,” Phys. Rev. Lett. , 038301 (2007). 10.1103/PhysRevLett.99.038301 17678335

[9] S. Hagen , F. Leyssner , D. Nandi , M. Wolf , and P. Tegeder , “ Reversible switching of tetra-tert-butyl-azobenzene on a Au(111) surface induced by light and thermal activation,” Chem. Phys. Lett. , 85 (2007). 10.1016/j.cplett.2007.07.005

[10] R. Schmidt , S. Hagen , D. Brete , R. Carley , C. Gahl , J. Dokić , P. Saalfrank , S. Hecht , P. Tegeder , and M. Weinelt , “ On the electronic and geometrical structure of the *trans*- and *cis*-isomer of tetra-*tert*-butyl-azobenzene on Au(111),” Phys. Chem. Chem. Phys. , 4488 (2010). 10.1039/b924409c 20407723

[11] E. McNellis , C. Bronner , and J. Meyer , “ Azobenzene versus 3,3′,5,5′-tetra-tert-butyl-azobenzene (TBA) at Au(111): characterizing the role of spacer groups,” Phys. Chem. Chem. Phys. , 6404 (2010). 10.1039/c001978j 20379594

[12] J. Osterwalder “Photoelectron spectroscopy and diffraction,” in *Surface and Interface Science*, edited by Wandelt K. ( Wiley-VCH, Weinheim, 2012), Vol. 1.

[13] R. S. Saiki , G. S. Herman , M. Yamada , J. Osterwalder , and C. S. Fadley , “ Structure of an unusual tilted state of Co on Fe(001) from x-ray photoelectron diffraction,” Phys. Rev. Lett. , 283 (1989). 10.1103/PhysRevLett.63.283 10041029

[14] M. Greif , L. Castiglioni , A. P. Seitsonen , S. Roth , J. Osterwalder , and M. Hengsberger , “ Photoelectron diffraction in the x-ray and ultraviolet regime: Sn-phthalocyanine on Ag(111),” Phys. Rev. B , 085429 (2013). 10.1103/PhysRevB.87.085429

[15] M. Greif , T. Nagy , M. Soloviov , L. Castiglioni , M. Hengsberger , M. Meuwly , and J. Osterwalder , “ Following the molecular motion of near-resonant excited CO on Pt(111): A simulated x-ray photoelectron diffraction study based on molecular dynamics calculations,” Struct. Dyn. , 059901 (2016). 10.1063/1.4958888 26798798 PMC4711632

[16] M. Greif , L. Kasmi , L. Castiglioni , M. Lucchini , L. Gallmann , U. Keller , J. Osterwalder , and M. Hengsberger , “ Access to phases of coherent phonon excitations by femtosecond ultraviolet photoelectron diffraction,” Phys. Rev. B , 054309 (2016). 10.1103/PhysRevB.94.054309

[17] M. Wolf and P. Tegeder , “ Reversible molecular switching at a metal surface: A case study of tetra-tert-butyl-azobenzene on Au(111),” Surf. Sci. , 1506 (2009). 10.1016/j.susc.2008.11.049

[18] D. Naumović , A. Stuck , T. Greber , J. Osterwalder , and L. Schlapbach , “ Full-hemispherical photoelectron diffraction data from Cu(001): Energy dependence and comparison with single-scattering-cluster simulations,” Phys. Rev. B , 7462 (1993). 10.1103/PhysRevB.47.7462 10004741

[19] H. Rau , “ AZO compounds,” in *Photochromism*, edited by Dürr H. and Bouas-Laurent H. ( Elsevier Science, Amsterdam, 2003), Chap. 4, pp. 165–192.

[20] S. Hagen , P. Kate , F. Leyssner , D. Nandi , M. Wolf , and P. Tegeder , “ Excitation mechanism in the photoisomerization of a surface-bound azobenzene derivative: Role of the metallic substrate,” J. Chem. Phys. , 164102 (2008). 10.1063/1.2997343 19045242

[21] M. Alemani , M. V. Peters , S. Hecht , K.-H. Rieder , F. Moresco , and L. Grill , “ Electric field-induced isomerization of azobenzene by STM,” J. Am. Chem. Soc. , 14446 (2006). 10.1021/ja065449s 17090013

[22] S. Hagen , “ Isomerization behaviour of photochromatic molecules in direct contact with noble metal surfaces,” Ph.D. thesis ( FU Berlin, 2009).

[23] A. Hemmi , H. Cun , S. Roth , J. Osterwalder , and T. Greber , “ Low cost photoelectron yield setup for surface process monitoring,” J. Vac. Sci. Technol. A , 023202 (2014). 10.1116/1.4866095

[24] T. Greber , O. Raetzo , T. J. Kreutz , P. Schwaller , W. Deichmann , E. Wetli , and J. Osterwalder , “ A photoelectron spectrometer for k-space mapping above the Fermi level,” Rev. Sci. Instrum. , 4549 (1997). 10.1063/1.1148429

[25] P. Baltzer , L. Karlsson , M. Lundqvist , and B. Wannberg , “ Resolution and signal-to-background enhancement in gas phase electron spectroscopy,” Rev. Sci. Instrum. , 2179 (1993). 10.1063/1.1143957

[26] A. Kramida , Yu. Ralchenko , J. Reader , and N. A. Team , *NIST Atomic Spectra Database (ver. 5.2)* ( National Institute of Standards and Technology, Gaithersburg, MD, 2014).

[27] M. J. Comstock , N. Levy , J. Cho , L. Berbil-Bautista , M. F. Crommie , D. A. Poulsen , and J. M. J. Fréchet , “ Measuring reversible photomechanical switching rates for a molecule at a surface,” Appl. Phys. Lett. , 123107 (2008). 10.1063/1.2901877

[28] S. Hagen , P. Kate , M. Peters , S. Hecht , M. Wolf , and P. Tegeder , “ Kinetic analysis of the photochemically and thermally induced isomerization of an azobenzene derivative on Au(111) probed by two-photon photoemission,” Appl. Phys. A , 253 (2008). 10.1007/s00339-008-4831-5

[29] F. J. García de Abajo , M. A. Van Hove , and C. S. Fadley , “ Multiple scattering of electrons in solids and molecules: A cluster-model approach,” Phys. Rev. B , 075404 (2001). 10.1103/PhysRevB.63.075404

[30] L. Despont , D. Naumovic , F. Clerc , C. Koitzsch , M. Garnier , F. G. de Abajo , M. van Hove , and P. Aebi , “ X-ray photoelectron diffraction study of Cu(111): Multiple scattering investigation,” Surf. Sci. , 380 (2006). 10.1016/j.susc.2005.10.038

[31] S. Hüfner , J. Osterwalder , T. Greber , and L. Schlapbach , “ Interpretation of substrate photoelectron diffraction,” Phys. Rev. B , 7350 (1990). 10.1103/PhysRevB.42.7350 9994875

[32] D. Friedman and C. Fadley , “ Final-state effects in photoelectron diffraction,” J. Electron. Spectrosc. Relat. Phenom. , 689 (1990). 10.1016/0368-2048(90)80191-C

[33] K.-M. Schindler , V. Fritzsche , M. C. Asensio , P. Gardner , D. E. Rickena , A. W. Robinson , A. M. Bradshaw , D. P. Woodruff , J. C. Conesa , and A. R. González-Elipe , “ Structural determination of a molecular adsorbate by photoelectron diffraction: Ammonia on Ni(111),” Phys. Rev. B , 4836 (1992). 10.1103/PhysRevB.46.4836 10004244

[34] M. Muntwiler , W. Auwärter , F. Baumberger , M. Hoesch , T. Greber , and J. Osterwalder , “ Determining adsorbate structures from substrate emission x-ray photoelectron diffraction,” Surf. Sci. , 125 (2001). 10.1016/S0039-6028(00)00928-6

[35] J. B. Pendry , “ Reliability factors for LEED calculations,” J. Phys. C: Solid State Phys. , 937 (1980). 10.1088/0022-3719/13/5/024

[36] K. Pearson , “ Note on regression and inheritance in the case of two parents,” Proc. R. Soc. London , 240 (1895). 10.1098/rspl.1895.0041

[37] J. Yeh and I. Lindau , “ Atomic subshell photoionization cross sections and asymmetry parameters: 1 ≤ *Z* ≤ 103,” At. Data Nucl. Data Tables , 1 (1985). 10.1016/0092-640X(85)90016-6

[38] M. Greif , L. Castiglioni , D. Becker-Koch , J. Osterwalder , and M. Hengsberger , “ Acquisition of photoelectron diffraction patterns with a two-dimensional wide-angle electron analyzer,” J. Electron. Spectrosc. Relat. Phenom. , 30 (2014). 10.1016/j.elspec.2014.08.007

